# The Emerging Role of Sperm-Associated Antigen 6 Gene in the Microtubule Function of Cells and Cancer

**DOI:** 10.1016/j.omto.2019.08.011

**Published:** 2019-09-10

**Authors:** Da-Fang Zheng, Qi Wang, Jing-Ping Wang, Zheng-Qi Bao, Shi-Wu Wu, Li Ma, Da-Min Chai, Z. Peter Wang, Yi-Sheng Tao

**Affiliations:** 1 Department of Pathology, The First Affiliated Hospital of Bengbu Medical College, Bengbu, Anhui 233030, China; 2 Department of Biochemistry and Molecular Biology, School of Laboratory Medicine, Bengbu Medical College, Bengbu, Anhui 233030, China; 3 Department of Pathology, Beth Israel Deaconess Medical Center, Harvard Medical School, Boston, MA 02215, USA

**Keywords:** SPAG6, microtubule, apoptosis, cancer, proliferation

## Abstract

Accumulated evidence shows that sperm-associated antigen 6 (SPAG6) gene has multiple biological functions. It maintains the normal function of a variety of cells including ciliary/flagellar biogenesis and polarization, neurogenesis, and neuronal migration. Moreover, SPAG6 is found to be critically involved in auditory transduction and the fibroblast life cycle. Furthermore, SPAG6 plays an essential role in immuno-regulation. Notably, SPAG6 has been demonstrated to participate in the occurrence and progression of a variety of human cancers. New evidence shows that SPAG6 gene regulates tumor cell proliferation, apoptosis, invasion, and metastasis. Therefore, in this review, we describe the physiological function and mechanism of SPAG6 in human normal cells and cancer cells. We also highlight that SPAG6 gene may be an effective biomarker for the diagnosis of human cancer. Taken together, targeting SPAG6 could be a novel strategy for the treatment of human diseases including cancer.

## Main Text

**Figure 1:**
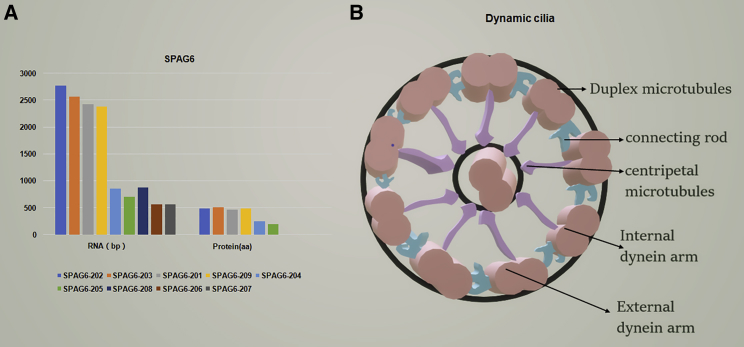
The Expression Level of SPAG6 and Its Location in the Centripetal Microtubules (A) The information about the RNA and the protein expressed by the SPAG6 gene, which has nine transcripts and only six proteins translated. (B) The cross section of the cilia, where SPAG6 expresses the centripetal microtubules.

The sperm-associated antigen 6 (SPAG6), also known as CT141, repro-sa-1, RP11-301N24.4, and pf16, was identified as a cancer-testis antigen (CTA) and is encoded by a gene located at chromosome 10p12.3.^1^, ^2^ Human SPAG6 has nine splicing variants: SPAG6-201, SPAG6-202, SPAG6-203, SPAG6-204, SPAG6-205, SPAG6-206, SPAG6-207, SPAG6-208, and SPAG6-209 (Figure 1). Murine SPAG6 also exists in the form of two isoforms, the parental SPAG6-BC061194 and its derivative, that are encoded by two homologous genes.^3^ The SPAG6 mRNA consists of 10 exons and 16 domains, whereas the protein contains eight consecutive WD repeats, which are known to mediate protein-protein interactions during brain development (Figure 1; Figure S1).^4^ Studies indicate that SPAG6 is a microtubule-associated protein that is essential for cytoskeleton formation. For instance, SPAG6 is present in the central organ protein of algae C1 microtubules and is a component of the central organ of the “9+2” axon,^5^ which is essential for ciliary and flagellar movement. In addition, it has also been detected in the inner ear hair cells, lung bronchial cells, ovarian epithelial cells, lymphocytes, and fibroblasts,^6^, ^7^ as well as in testicular germ cell tumors, hematological malignancies, breast and lung cancer, and bladder cancer.^1^, ^8^, ^9^ In this review, we focus on the function of SPAG6 in various cells and the molecular mechanism involved in SPAG6-mediated tumorigenesis and progression. In addition, we also discuss its potential role in tumor diagnosis and treatment.

### Physiological Functions of SPAG6

#### Ciliary/Flagellar Biogenesis and Polarization

As a microtubule-binding protein, SPAG6 plays an important role in the biogenesis of motility structures like cilia and flagella,^10^ although the mechanisms are likely different.^10^ The motile cilia are formed after the assembly of nine microtubules with two linear centripetal microtubules,^11^ and *spag6* knockout mice ciliary drift because of the loss of ciliary pair of central tubulin.^12^ Zhang et al.^13^ showed that *spag6*^*−/−*^ mice had intact cilia on their trachea epithelial cells with the “9+2” axoneme structure, whereas another study found that the bronchial epithelial cell ciliary function was defective in these mice.^7^ Furthermore, the spermatozoan flagella of the *spag6*^*−/−*^ mice have the “9+1” or “9+0” axoneme arrangement.^5^ SPAG6 can affect the function of these motility structures by altering the apical microtubule network,^7^ which lies upstream of the planar cell polarity (PCP) signaling cascade^14^, ^15^ and controls the tissue-level polarity of tracheal epithelial cells.^16^ The PCP proteins Prickle2, Dishevelled1, Dvl2, Vangl1, and Vangl2 localize asymmetrically to the tracheal epithelial cell cortex,^17^ and disruption of these proteins obliterates rotational polarity of these cells.^18^, ^19^, ^20^ The distribution of the PCP proteins is also disrupted in the *spag6*^*−/−*^ mice, which impairs the sub-apical microtubule stability and polarization of basal bodies in the relevant cells.^7^ Furthermore, Teves et al.^6^ detected arrhythmic and reduced ciliary beat in the tracheal epithelial cells of *spag6*^*−/−*^ mice, along with disarrayed and significantly sparse cilia. In addition, the orientation of the basal feet that determine axoneme orientation was random,^7^ and the levels of mucin, α-tubulin, and Vangl2 were significantly decreased.^7^ Taken together, SPAG6 regulates ciliary/flagellar motility, and ciliogenesis, axoneme orientation, and tracheal epithelial cell polarity are adversely affected in its absence.^7^

#### Neurogenesis and Neuronal Migration

Studies have increasingly shown a critical role of SPAG6 in neurological functions.^19^ Co-localization of SPAG6 and microtubules has been observed in various cells lines,^4^, ^21^, ^22^ and SPAG6 is overexpressed in the chicken embryonic spinal cord.^23^ The human SPAG6 and the *Chlamydomonas* pf16 can bind to the centrosome proteins Wdr62 and LIS1 through their contiguous WD repeats,^24^, ^25^ indicating their involvement in Wdr62-mediated mitotic spindle regulation and neurogenesis.^19^ Mitchell et al.^16^ proposed that SPAG6 likely affects neuronal migration by targeting microtubules, which control centrosome movement and soma during neuronal migration,^26^, ^27^ indicating that SPAG6 also plays a role in neural development.^28^ The two modes of neuronal migration, known as soma translocation and radial glia-dependent locomotion, need coordinated cytoskeletal remodeling.^29^ Yan et al.^28^ showed that overexpression of SPAG6 delayed the rate of neuronal migration, branching, and elongation, indicating that it can stabilize the microtubules and prevent remodeling.^28^ Another study found fewer microtubules and actin filaments, in addition to other abnormal membranous structures, in the spiral ganglion neurons (SGNs) of *spag6*^*−/−*^ mice.^30^ SPAG6 is localized in the peri-nuclear cytoplasm of wild-type SGNs and decreased in the *spag6*^*−/−*^ mice by perinatal day 30.^30^ Furthermore, RNAi-mediated inhibition of *spag6* in multi-ciliated mouse ependymal cells completely abolished the central pair microtubules and ciliary localization of SPAG6, resulting in rotational ciliary movement.^15^ Finally, the *spag6* promoter is hypermethylated during the differentiation of human embryonic stem cells into neural progenitor/stem cells (NPCs) *in vitro*, indicating that SPAG6 levels are modulated during neurogenesis.^31^

#### Auditory Transduction

SPAG6 is also essential for the mechano-sensory function of the cylindrical outer hair cells (OHCs) in the organ of Corti, which is the receptor organ for hearing in the mammalian cochlea.^32^ Evidence suggests that patients with primary ciliated hair cells of Corti often have hearing impairment simultaneously.^33^ The normal physiological function of the hair cells on the Corti is to maintain hearing production by changing their length and stiffness by a recognized molecular click that drives from a putative molecular motor designated prestin,^34^, ^35^ and dyskinesia happens to the cilia of the hair cells missing spag6.^36^ Wang et al.^37^ were the first to detect SPAG6 in the OHCs, and they found that SPAG6 and the microtubule-associated protein MAP1S bound to and stabilized prestin,^38^ which is essential for maintaining the normal function of the OHCs.^37^ In their study, immunofluorescent staining of SPAG6 and prestin shows expression of SPAG6 in the lateral wall of OHCs, and in the cuticular plate in OHCs, while prestin located in the cuticular plate of OHCs.^37^ Prestin protein and mRNA levels were decreased in SPAG6 defect mice.^37^ Li et al.^39^ studied the relationship between SPAG6 and otitis media, and they have proved that SPAG6 regulates cilia/basal body polarity through the PCP-dependent mechanisms that not only regulate cilium location or orientation, but also regulate basal body docking to the apical surface during ciliogenesis in the middle ear and Eustachian tubes.^40^, ^41^ Li et al.’s^39^ study showed that the orientation of the ciliary basal feet was random in the middle ear epithelial cells of SPAG6-deficient mice. Frizzled class receptor 6 (FZD6), a core planar cell polarity (PCP) protein, is a mediator of the Wnt pathway and plays a critical role in differentiation and organism development.^42^ FZD6 does not have a polarized distribution in the mutant tympanic ciliary epithelium,^39^ indicating that FZD6 protein location was altered by inactivation of SPAG6 in the middle ear, and that planar polarity of the middle ear was affected,^39^ which is in keeping with the report that FZD6 knockout mice have developmental disorders in the planar polarity of the OHC and neural tube closure,^43^ and “9+2” axonemes of cilia were conserved; also, the orientation of central microtubules was not uniform,^39^ indicating that as in the brain and lungs, SPAG6 mutation affects the polarity of the middle ear epithelium, with the gross structure of the “9+2” axonemes being conserved.^7^, ^13^ It is interesting to note that other research has shown that the SPAG6 gene has not been associated with hair cell differentiation.^44^ There are also some experimental observations worth exploring. In one study, the researchers found that SPAG6-deficient mice exhibited abnormal behaviors, such as logy motion and continuous head tossing, and fur loss occurred in comparison with their corresponding SPAG6 overexpression and SPAG6 normal expression littermates.^37^

#### Immuno-regulation

The immune synapse has the same centrosome nucleation mechanism as the cilia and, therefore, similar functions.^5^ Intraflagellar transport protein 20 (IFT20), a protein involved in ciliary formation like SPAG6, also plays an important role in the immune synapse between the T cell receptor (TCR) and antigen-presenting cells (APCs) or target cells.^45^, ^46^ This led to the speculation that SPAG6 may also be involved in the lymphatic system. de la Roche et al.^47^ showed that the TCR immune synapses function as cilia at the junction of T cells and APCs/target cells. In addition, SPAG6 is expressed in both primary and secondary lymphoid tissues, and its deletion ruptured immune synapses because of the absence of centrosome polarization and actin clearance in the synaptic cleft. During the homologous recognition between APCs and effector cells, the centrosomes, actin, Golgi bodies, and secretory vesicles are reoriented at the immune synapse, allowing receptor/ligand interactions and targeted release of cytokines.^47^ In addition, during targeted killing of effector cells, the docking between centrosomes and the effector cells’ synaptic membrane also occurs in the same direction, thereby effectively forming the synaptic gap for targeted lysozyme release.^46^, ^48^ Based on these findings, it is reasonable to surmise that SPAG6-mediated polarization of synaptic centrosomes and the removal of actin are essential for T cell cytotoxicity.^5^

#### Fibroblast Life Cycle

SPAG6 has been implicated in fibroblast growth, morphology, migration, and ciliary movement.^7^ Li et al.^7^ found that mouse embryonic fibroblasts (MEFs) lacking the *Spag6* gene had greater surface area, abnormal morphology, slower proliferation rates, and less motility compared with the wild-type MEFs, and these defects were alleviated upon forced expression of exogenous SPAG6. In addition, the *Spag6*^*−/−*^ MEFs also showed impaired surface adhesion, significant reduction in primary cilia, and even presence of multiple cilia in some cells because of non-polarized F-actin. Multiple centrosomes were also observed in the cytoplasm of the *Spag6*^*−/−*^ MEFs,^7^ along with increased levels of the microtubule stability marker acetylated tubulin in *spag6*^*+/+*^ MEFS.^7^ Consistent with this, these defective MEFs were highly sensitive to paclitaxel, a known microtubule stabilizer.^7^ Also, microtubule acetylation is positively correlated with transfection efficiency^49^ and was low in the *Spag6*^*−/−*^ MEFs.^7^ Therefore, Li et al.^7^ also proposed the functional mechanism of SPAG6 regulating MEF cells; that is, the function of SPAG6 itself may be a tubulin acetyltransferase or deacetylase inhibitor. Re-expression of spag6 with adenovirus vector rescued abnormal morphology and reduced levels of acetylated tubulin.^7^ However, the loss of MEFs acetylated tubulin expression in spag6 can damage the function of microtubules and cause the phenotype changes of cell growth, migration, adhesion, division, and ciliogonium.^7^ In one study, Glu-tubulin is released from C-terminal tyrosine of alpha tubulin by tubulin carboxypeptidase,^50^ and its expression level was modestly increased when SPAG6 expression level was increased. Given the fact that SPAG6 is also upregulated in cancers.^51^ Li et al.^7^ proposed that high SPAG6 expression in these cancer cells may also increase Glu-tubulin expression.

#### Regulation of SPAG6

Spef1, an evolutionary conserved molecular binding protein, binds microtubules (MTs) through its amino-terminal calponin-homologous domain, and forms homologous dimer and stabilizes microtubules through its C-terminal curling helix region; Spef1 also enables mammalian ciliary central apparatus formation.^15^ Cilia signal of Spef1 was weakened in multiciliated ependymal cells (mEPCs) treated with cisplatin, and cilia signal of Spag6 was also weakened.^15^ One study showed that the PF6, SPAG6, and PF20 proteins form an interactive network, which presumably links the central apparatus microtubules and their projections into a functional unit, and their gene promoters share a common transcription factor binding site.^4^, ^52^ Although these proteins do not regulate the primary ciliary function of Madin-Darby canine kidney (MDCK) cells, they modulate the expression of nine peri-ciliary and cilia-associated proteins.^52^ SOX5 is a transcription factor, which has a homology to the region of the testis-determining factor, sex-determining region of the Y (SRY).^53^ The SRY-related high mobility group box or SOX family of transcription factors regulates the development of retinal, muscle, endothelial, epidermal, intestinal, lymphoid, and cartilaginous tissues,^53^ and a 48-kDa SOX5 protein (S-SOX5) that is encoded by a SOX5 gene in particular has been associated with SPAG6 regulation.^54^ Forkhead box J1 (FOXJ1) is a transcriptional factor that participates in several cellular processes including immune homeostasis and tumorigenesis.^55^ S-SOX5 and FOXJ1 synergistically upregulate *spag6* promoter activity,^56^ and therefore may mediate cilia/flagella biogenesis by globally regulating the expression of axon protein-encoding genes.^52^ Pf20 is co-expressed with SPAG6 in microtubules during polymerization, and transient scrotal heat can affect SPAG6 expression.^57^

#### Pathological Function of SPAG6

Studies have increasingly shown a critical role of SPAG6 in tumor progression, especially in hematological malignancies that harbor higher SPAG6 levels compared with solid tumors.^58^ As a testicular-specific and cytoskeletal protein,^59^ genetic deficiency of SPAG6 is associated with inherited diseases such as immotile-cilia syndrome, situs inversus totalis, hydrocephalus, anosmia, and retinitis pigmentosa, as well as male and female infertility.^60^ The CGI promoters of *spag6* and several other genes were methylated in a neuroblastoma (NBLs) cell line, resulting in the silencing of downstream genes.^61^ In addition, *spag6* and *c20orf85* were the only differentially expressed genes between normal fallopian tube epithelia (FTE) and high-grade serous ovarian cancer (HGSOC) samples.^62^ Kitchen et al.^21^ also found frequent methylation of the *spag6* promoter in bladder cancer tissues. Global gene expression analysis of the bronchial epithelium showed high levels of ciliogenesis-associated genes such as *dnai2*, *spag6*, *asp*, and *foxj1*,^22^ of which *spag6* is involved in the transcriptional regulation of DNA methylation in non-small-cell lung cancer.^63^ In addition, *spag6* and *l1td1* were methylated in non-small-cell lung cancer (NSCLC) primary tumors as opposed to the non-malignant lung tissues, which regulated the transcription of different genes.^63^ The recurrent t(10;11) (p12;Q14) translocation in acute myeloid leukemia (AML), acute lymphoblastic leukemia (ALL), and malignant lymphoma results in the fusion of the genes encoding the putative zinc-finger transcription factor AF10 and the clathrin assembly lymphoid myeloid leukemia protein (CALM).^64^ Transcriptional analysis of 20 CALM/AF10 fusion-positive leukemia samples showed a significant upregulation of *spag6* near the fusion site,^25^ which was, however, absent in leukemic cells isolated from CALM/AF10 transplanted mouse models that lacked the specific translocation.^25^

Monitoring of minimal residual disease (MRD) has become a powerful diagnostic tool for AML.^9^ Steinbach et al.^9^ identified SPAG6, along with chemokine C-C motif ligand 23 (CCL23), mesothelin (MSLN), suppression of tumorigenicity 18 (ST18), G antigen family D2 (GAGED2), Wilms tumor gene (WT1), and preferentially expression antigen in melanoma 9 (PRAME9), as an MRD marker in AML monitoring. In a follow-up sample analysis of 145 cases, these seven genes were overexpressed in the leukemia cells of 52 children with AML and 25 recovered patients and were found in the test results of patients in complete remission.^9^ Thus, SPAG6 is a potential marker for predicting MRD in children with acute myelopathies.^9^ Yang et al.^65^ showed that SPAG6 silencing inhibited the growth of malignant myeloid SKM-1 and K562 cell lines by activating p53, phosphatase and tensin homolog (PTEN), and caspase-3, -8, and -9, and triggered the caspase-dependent apoptotic cascade. Therefore, SPAG6 is a potential prognostic factor in hematological malignancies. One study showed that SPAG6 may also be involved in TNF-related apoptosis-inducing ligand (TRAIL)-induced apoptosis.^66^ Both death receptor and mitochondria-triggered apoptosis are culminated via caspases.^26^, ^67^ Jiang et al.^68^ also found that *spag*6 gene silencing in SKM-1 cells activated the PTEN/phosphatidylinositol 3-kinase (PI3K)/AKT signaling pathway by increasing PTEN phosphorylation and decreasing that of AKT, leading to apoptosis and differentiation of the leukemia cells. DNA methyltransferase 1 (DNMT1) is a cytosine methylase that is important for normal mammalian development, and DNMT1 mutations are correlated with human cancer.^69^ In addition, *spag6* knockdown was also associated with downregulation of DNMT1, suggesting that SPAG6 may indirectly control the expression of PTEN through DNA methylation.^70^ The anti-proliferative effects of *spag6* knockdown were mediated via upregulation of the cyclin-dependent kinase inhibitor p27^Kip1^ and the AKT/forkhead box protein O (FOXO) pathway.^68^ The tumor suppressor p27^Kip1^ induces G1-S phase arrest by inhibiting the cyclin E-CDK2 and cyclin A-CDK2 complexes, which are abnormally downregulated in multiple tumors.^29^, ^71^ The *spag6* knockdown in SKM-1 cells exhibited G1 phase arrest, p27^Kip1^ upregulation, and cyclin E1 and CDK2 downregulation (Table 1).^68^

**Table 1:** Roles of spag6 in Various Types of Cells and Organs

Cell Types	Functions	Target	Reference
Tracheal epithelial cells	cilia density, tissue-level polarity, axoneme orientation, beat frequency	core PCP genes	7, 13, 14, 15, 16
Neurons	migration neurogenesis and differentiation	cytoskeletal hypermethylation of the SPAG6 promoter CpG	19, 24, 27, 28, 30, 73
Outer hair cells	mechanosensory function, hearing generation	prestin; PCP proteins; FZD6	34, 35, 38, 39
Immune system	synapse disruption maintains T cell cytotoxicity, antigen presentation	centrosome	13, 33, 46, 47
Fibroblast cell	growth, morphology, migration, and ciliogenesis	acetylated tubulin microtubule	7, 51
Neuroblastoma	diagnosis	genes were methylated	^61^
MRD/ALL/MDS	apoptosis	p53 caspase PTEN/PI3K/AKT TRAIL	21, 22, 25, 29, 61, 62, 64, 68
Bladder cancer	diagnosis	promoter-related CpG island methylation	^21^
HGSOC/FTE	diagnosis	not detected	^62^
NSCLC	diagnosis	DNA methylation of SPAG6	^63^

MDS, myelodysplastic syndrome.

### Conclusions

**Figure 2:**
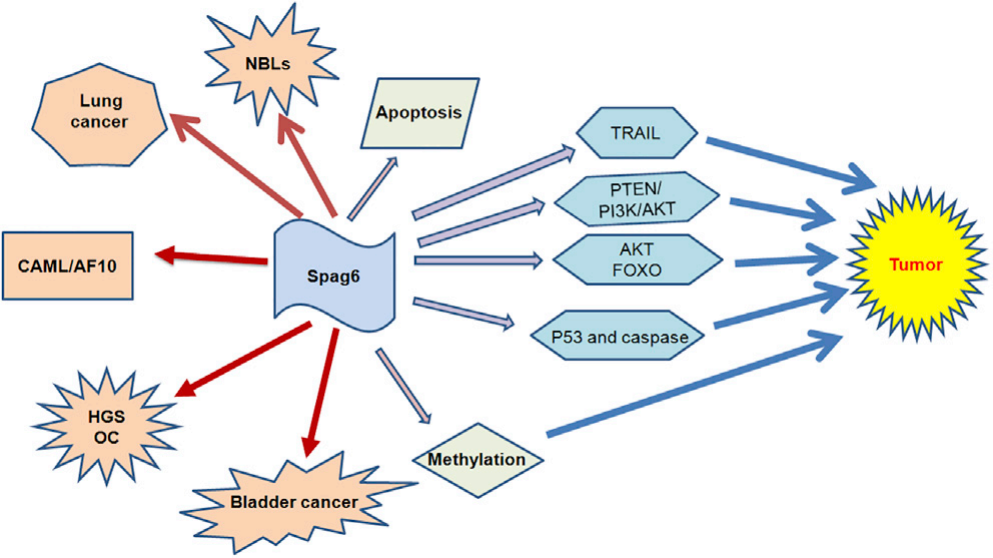
SPAG6 Regulates Multiple Functions in Various Cell Types SPAG6 controls numerous cellular signaling pathways to participate in tumorigenesis including lung cancer and bladder cancer.

**Figure 3:**
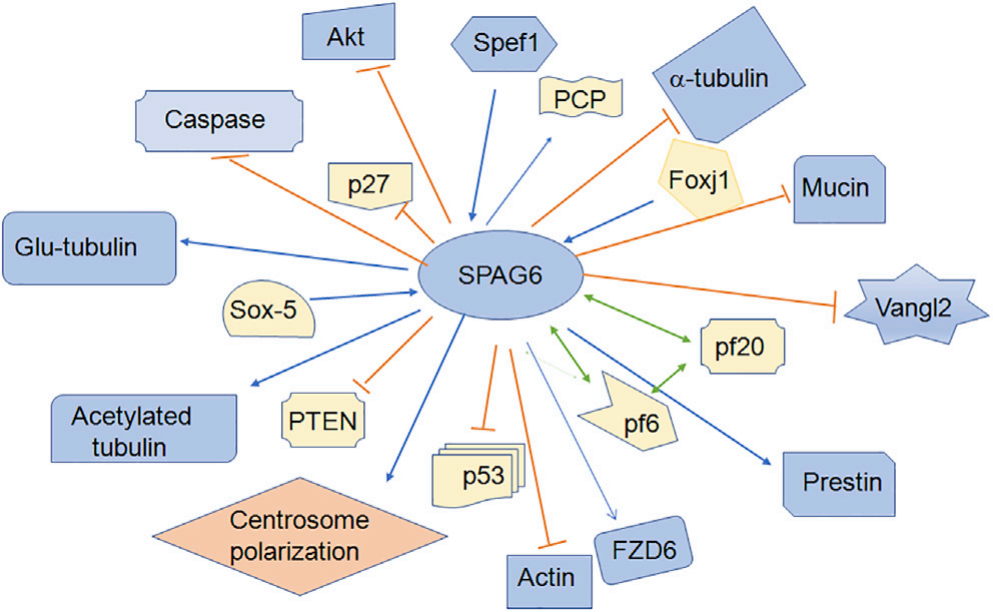
SPAG6 Exerts Its Multiple Functions via Different Molecular Mechanisms SPAG6 targets numerous genes to participate in its biological function. SPAG6 is regulated by SOX5, Spef1, and Foxj1.

SPAG6 regulates multiple functions in various cell types, and its loss can lead to pathophysiological conditions via different molecular mechanisms (Figures 2 and 3). The differential expression of SPAG6 in tumor tissues indicates its diagnostic potential. Furthermore, SPAG6 is also a promising anti-cancer therapeutic target because its absence can stabilize the microtubules,^30^, ^45^ enhance the effects of apoptosis-inducing drugs,^65^ and induce cell-cycle arrest.^64^ SPAG6 plays a major role in regulating the microtubule/cytoskeletal system,^7^ as well as the associated cellular functions, by binding to microtubules.^5^ Studies also show a positive correlation between gluten-tubulin and SPAG6 overexpression in malignant tumors.^51^ Microtubule-actin interactions are critical for cell movement and morphogenesis,^65^ although it remains to be determined whether SPAG6 affects the actin filaments directly or indirectly through the microtubules. SPAG6 has also been implicated in immune deficiencies,^5^ because its absence disrupts the effector-target cell synapses that are necessary for an effective immune response. Recent studies show a possible role of SPAG6 in microtubule assembly and cell migration. Various factors are involved in SPAG6 regulation, including the newly ascertained transcription factors GATA-3 and Pou4f3.^36^, ^72^ There are several key aspects of SPAG6 biology that need to be studied further. For example, more data are required to support the diagnostic and therapeutic possibilities of SPAG6 in cancer, in addition to identifying the pathologically relevant *spag6* mutations in cancer patients. Furthermore, the upstream regulatory factors and downstream targets of SPAG6, and the pro-tumorigenic factors that synergize with SPAG6 need to be identified. Tissue-specific knockout or transgenic mouse models can greatly elucidate the role of SPAG6 in cancer development.

## Author Contributions

D.-F.Z., Q.W., J.-P.W., Z.-Q.B., S.-W.W., and L.M. searched the literature and made the figures and tables. D.-F.Z. and D.-M.C. wrote the manuscript. Z.P.W. and Y.-S.T. edited the manuscript. All authors read and approved the final manuscript.

## Conflicts of Interest

The authors declare no competing interests.

## Supplemental Information

Document S1. Figure S1

Document S2. Article plus Supplemental Information

## References

[1] Siliņa K., Zayakin P., Kalniņa Z., Ivanova L., Meistere I., Endzeliņš E., Abols A., Stengrēvics A., Leja M., Ducena K. (2011). Sperm-associated antigens as targets for cancer immunotherapy: expression pattern and humoral immune response in cancer patients. J. Immunother..

[2] Neilson L.I., Schneider P.A., Van Deerlin P.G., Kiriakidou M., Driscoll D.A., Pellegrini M.C., Millinder S., Yamamoto K.K., French C.K., Strauss J.F. (1999). cDNA cloning and characterization of a human sperm antigen (SPAG6) with homology to the product of the Chlamydomonas PF16 locus. Genomics.

[3] Qiu H., Gołas A., Grzmil P., Wojnowski L. (2013). Lineage-specific duplications of Muroidea Faim and Spag6 genes and atypical accelerated evolution of the parental Spag6 gene. J. Mol. Evol..

[4] Zhang Z., Sapiro R., Kapfhamer D., Bucan M., Bray J., Chennathukuzhi V., McNamara P., Curtis A., Zhang M., Blanchette-Mackie E.J., Strauss J.F. (2002). A sperm-associated WD repeat protein orthologous to Chlamydomonas PF20 associates with Spag6, the mammalian orthologue of Chlamydomonas PF16. Mol. Cell. Biol..

[5] Cooley L.F., El Shikh M.E., Li W., Keim R.C., Zhang Z., Strauss J.F., Zhang Z., Conrad D.H. (2016). Impaired immunological synapse in sperm associated antigen 6 (SPAG6) deficient mice. Sci. Rep..

[6] Teves M.E., Zhang Z., Costanzo R.M., Henderson S.C., Corwin F.D., Zweit J., Sundaresan G., Subler M., Salloum F.N., Rubin B.K., Strauss J.F. (2013). Sperm-associated antigen-17 gene is essential for motile cilia function and neonatal survival. Am. J. Respir. Cell Mol. Biol..

[7] Li W., Mukherjee A., Wu J., Zhang L., Teves M.E., Li H., Nambiar S., Henderson S.C., Horwitz A.R., Strauss J.F. (2015). Sperm Associated Antigen 6 (SPAG6) Regulates Fibroblast Cell Growth, Morphology, Migration and Ciliogenesis. Sci. Rep..

[8] An T., Gong Y., Li X., Kong L., Ma P., Gong L., Zhu H., Yu C., Liu J., Zhou H. (2017). USP7 inhibitor P5091 inhibits Wnt signaling and colorectal tumor growth. Biochem. Pharmacol..

[9] Steinbach D., Schramm A., Eggert A., Onda M., Dawczynski K., Rump A., Pastan I., Wittig S., Pfaffendorf N., Voigt A. (2006). Identification of a set of seven genes for the monitoring of minimal residual disease in pediatric acute myeloid leukemia. Clin. Cancer Res..

[10] Lee L., Campagna D.R., Pinkus J.L., Mulhern H., Wyatt T.A., Sisson J.H., Pavlik J.A., Pinkus G.S., Fleming M.D. (2008). Primary ciliary dyskinesia in mice lacking the novel ciliary protein Pcdp1. Mol. Cell. Biol..

[11] Coutton C., Martinez G., Kherraf Z.E., Amiri-Yekta A., Boguenet M., Saut A., He X., Zhang F., Cristou-Kent M., Escoffier J. (2019). Bi-allelic Mutations in ARMC2 Lead to Severe Astheno-Teratozoospermia Due to Sperm Flagellum Malformations in Humans and Mice. Am. J. Hum. Genet..

[12] Sapiro R., Kostetskii I., Olds-Clarke P., Gerton G.L., Radice G.L., Strauss J.F. (2002). Male infertility, impaired sperm motility, and hydrocephalus in mice deficient in sperm-associated antigen 6. Mol. Cell. Biol..

[13] Zhang Z., Tang W., Zhou R., Shen X., Wei Z., Patel A.M., Povlishock J.T., Bennett J., Strauss J.F. (2007). Accelerated mortality from hydrocephalus and pneumonia in mice with a combined deficiency of SPAG6 and SPAG16L reveals a functional interrelationship between the two central apparatus proteins. Cell Motil. Cytoskeleton.

[14] Shimada Y., Yonemura S., Ohkura H., Strutt D., Uemura T. (2006). Polarized transport of Frizzled along the planar microtubule arrays in Drosophila wing epithelium. Dev. Cell.

[15] Zheng J., Liu H., Zhu L., Chen Y., Zhao H., Zhang W., Li F., Xie L., Yan X., Zhu X. (2019). Microtubule-bundling protein Spef1 enables mammalian ciliary central apparatus formation. J. Mol. Cell Biol..

[16] Mitchell B., Stubbs J.L., Huisman F., Taborek P., Yu C., Kintner C. (2009). The PCP pathway instructs the planar orientation of ciliated cells in the Xenopus larval skin. Curr. Biol..

[17] Vladar E.K., Bayly R.D., Sangoram A.M., Scott M.P., Axelrod J.D. (2012). Microtubules enable the planar cell polarity of airway cilia. Curr. Biol..

[18] Fischer D., Laiho A., Gyenesei A., Sironen A. (2015). Identification of Reproduction-Related Gene Polymorphisms Using Whole Transcriptome Sequencing in the Large White Pig Population. G3 (Bethesda).

[19] Bogoyevitch M.A., Yeap Y.Y., Qu Z., Ngoei K.R., Yip Y.Y., Zhao T.T., Heng J.I., Ng D.C. (2012). WD40-repeat protein 62 is a JNK-phosphorylated spindle pole protein required for spindle maintenance and timely mitotic progression. J. Cell Sci..

[20] Tissir F., Qu Y., Montcouquiol M., Zhou L., Komatsu K., Shi D., Fujimori T., Labeau J., Tyteca D., Courtoy P. (2010). Lack of cadherins Celsr2 and Celsr3 impairs ependymal ciliogenesis, leading to fatal hydrocephalus. Nat. Neurosci..

[21] Kitchen M.O., Bryan R.T., Haworth K.E., Emes R.D., Luscombe C., Gommersall L., Cheng K.K., Zeegers M.P., James N.D., Devall A.J. (2015). Methylation of HOXA9 and ISL1 Predicts Patient Outcome in High-Grade Non-Invasive Bladder Cancer. PLoS ONE.

[22] Lonergan K.M., Chari R., Deleeuw R.J., Shadeo A., Chi B., Tsao M.S., Jones S., Marra M., Ling V., Ng R. (2006). Identification of novel lung genes in bronchial epithelium by serial analysis of gene expression. Am. J. Respir. Cell Mol. Biol..

[23] Hamada T., Teraoka M., Imaki J., Ui-Tei K., Ladher R.K., Asahara T. (2010). Gene expression of Spag6 in chick central nervous system. Anat. Histol. Embryol..

[24] Hu X., Yan R., Cheng X., Song L., Zhang W., Li K., Zhao S. (2016). The function of sperm-associated antigen 6 in neuronal proliferation and differentiation. J. Mol. Histol..

[25] Mulaw M.A., Krause A., Deshpande A.J., Krause L.F., Rouhi A., La Starza R., Borkhardt A., Buske C., Mecucci C., Ludwig W.D. (2012). CALM/AF10-positive leukemias show upregulation of genes involved in chromatin assembly and DNA repair processes and of genes adjacent to the breakpoint at 10p12. Leukemia.

[26] Fulda S., Debatin K.M. (2006). Extrinsic versus intrinsic apoptosis pathways in anticancer chemotherapy. Oncogene.

[27] Wu Q., Liu J., Fang A., Li R., Bai Y., Kriegstein A.R., Wang X., Nguyen L., Hippenmeyer S. (2014). The dynamics of neuronal migration. Cellular and Molecular Control of Neuronal Migration.

[28] Yan R., Hu X., Zhang Q., Song L., Zhang M., Zhang Y., Zhao S. (2015). Spag6 negatively regulates neuronal migration during mouse brain development. J. Mol. Neurosci..

[29] Zeng W., Dai H., Yan M., Cai X., Luo H., Ke M., Liu Z. (2017). Decitabine-Induced Changes in Human Myelodysplastic Syndrome Cell Line SKM-1 Are Mediated by FOXO3A Activation. J. Immunol. Res..

[30] Li X., Xu L., Sun G., Wu X., Bai X., Li J., Strauss J.F., Zhang Z., Wang H. (2017). Spag6 Mutant Mice Have Defects in Development and Function of Spiral Ganglion Neurons, Apoptosis, and Higher Sensitivity to Paclitaxel. Sci. Rep..

[31] Shen Y., Chow J., Wang Z., Fan G. (2006). Abnormal CpG island methylation occurs during in vitro differentiation of human embryonic stem cells. Hum. Mol. Genet..

[32] Schwander M., Kachar B., Müller U. (2010). Review series: The cell biology of hearing. J. Cell Biol..

[33] Janke C., Bulinski J.C. (2011). Post-translational regulation of the microtubule cytoskeleton: mechanisms and functions. Nat. Rev. Mol. Cell Biol..

[34] Zheng J., Shen W., He D.Z.Z., Long K.B., Madison L.D., Dallos P. (2000). Prestin is the motor protein of cochlear outer hair cells. Nature.

[35] Yu N., Zhu M.L., Zhao H.B. (2006). Prestin is expressed on the whole outer hair cell basolateral surface. Brain Res..

[36] Gross J., Stute K., Fuchs J., Angerstein M., Amarjargal N., Mazurek B. (2011). Effects of retinoic acid and butyric acid on the expression of prestin and Gata-3 in organotypic cultures of the organ of corti of newborn rats. Dev. Neurobiol..

[37] Wang J., Li X., Zhang Z., Wang H., Li J. (2015). Expression of prestin in OHCs is reduced in Spag6 gene knockout mice. Neurosci. Lett..

[38] Bai J.P., Surguchev A., Ogando Y., Song L., Bian S., Santos-Sacchi J., Navaratnam D. (2010). Prestin surface expression and activity are augmented by interaction with MAP1S, a microtubule-associated protein. J. Biol. Chem..

[39] Li X., Xu L., Li J., Li B., Bai X., Strauss J.F., Zhang Z., Wang H. (2014). Otitis media in sperm-associated antigen 6 (Spag6)-deficient mice. PLoS ONE.

[40] Park T.J., Mitchell B.J., Abitua P.B., Kintner C., Wallingford J.B. (2008). Dishevelled controls apical docking and planar polarization of basal bodies in ciliated epithelial cells. Nat. Genet..

[41] Bayly R., Axelrod J.D. (2011). Pointing in the right direction: new developments in the field of planar cell polarity. Nat. Rev. Genet..

[42] Corda G., Sala A. (2017). Non-canonical WNT/PCP signalling in cancer: Fzd6 takes centre stage. Oncogenesis.

[43] Wang Y., Guo N., Nathans J. (2006). The role of Frizzled3 and Frizzled6 in neural tube closure and in the planar polarity of inner-ear sensory hair cells. J. Neurosci..

[44] Yoon H., Lee D.J., Kim M.H., Bok J. (2011). Identification of genes concordantly expressed with Atoh1 during inner ear development. Anat. Cell Biol..

[45] Finetti F., Patrussi L., Masi G., Onnis A., Galgano D., Lucherini O.M., Pazour G.J., Baldari C.T. (2014). Specific recycling receptors are targeted to the immune synapse by the intraflagellar transport system. J. Cell Sci..

[46] Stinchcombe J., Bossi G., Griffiths G.M. (2004). Linking albinism and immunity: the secrets of secretory lysosomes. Science.

[47] de la Roche M., Ritter A.T., Angus K.L., Dinsmore C., Earnshaw C.H., Reiter J.F., Griffiths G.M. (2013). Hedgehog signaling controls T cell killing at the immunological synapse. Science.

[48] Kloc M., Maffei A. (2014). Target-specific properties of thalamocortical synapses onto layer 4 of mouse primary visual cortex. J. Neurosci..

[49] Vaughan E.E., Geiger R.C., Miller A.M., Loh-Marley P.L., Suzuki T., Miyata N., Dean D.A. (2008). Microtubule acetylation through HDAC6 inhibition results in increased transfection efficiency. Mol. Ther..

[50] Contin M.A., Sironi J.J., Barra H.S., Arce C.A. (1999). Association of tubulin carboxypeptidase with microtubules in living cells. Biochem. J..

[51] Whipple R.A., Matrone M.A., Cho E.H., Balzer E.M., Vitolo M.I., Yoon J.R., Ioffe O.B., Tuttle K.C., Yang J., Martin S.S. (2010). Epithelial-to-mesenchymal transition promotes tubulin detyrosination and microtentacles that enhance endothelial engagement. Cancer Res..

[52] Horowitz E., Zhang Z., Jones B.H., Moss S.B., Ho C., Wood J.R., Wang X., Sammel M.D., Strauss J.F. (2005). Patterns of expression of sperm flagellar genes: early expression of genes encoding axonemal proteins during the spermatogenic cycle and shared features of promoters of genes encoding central apparatus proteins. Mol. Hum. Reprod..

[53] Donner A.L., Episkopou V., Maas R.L. (2007). Sox2 and Pou2f1 interact to control lens and olfactory placode development. Dev. Biol..

[54] Zhang L., Liu Y., Li W., Zhang Q., Li Y., Liu J., Min J., Shuang C., Song S., Zhang Z. (2017). Transcriptional regulation of human sperm-associated antigen 16 gene by S-SOX5. BMC Mol. Biol..

[55] Peng S.L. (2006). Interactions of Fox proteins with inflammatory transcription-factor pathways. Expert Rev. Clin. Immunol..

[56] Kiselak E.A., Shen X., Song J., Gude D.R., Wang J., Brody S.L., Strauss J.F., Zhang Z. (2010). Transcriptional regulation of an axonemal central apparatus gene, sperm-associated antigen 6, by a SRY-related high mobility group transcription factor, S-SOX5. J. Biol. Chem..

[57] Rao M., Xia W., Yang J., Hu L.X., Hu S.F., Lei H., Wu Y.Q., Zhu C.H. (2016). Transient scrotal hyperthermia affects human sperm DNA integrity, sperm apoptosis, and sperm protein expression. Andrology.

[58] Barretina J., Caponigro G., Stransky N., Venkatesan K., Margolin A.A., Kim S., Wilson C.J., Lehár J., Kryukov G.V., Sonkin D. (2012). The Cancer Cell Line Encyclopedia enables predictive modelling of anticancer drug sensitivity. Nature.

[59] Chen W., Liu Y.X., Jiang G.F. (2015). De novo Assembly and Characterization of the Testis Transcriptome and Development of EST-SSR Markers in the Cockroach Periplaneta americana. Sci. Rep..

[60] Afzelius B.A. (2004). Cilia-related diseases. J. Pathol..

[61] Abe M., Watanabe N., McDonell N., Takato T., Ohira M., Nakagawara A., Ushijima T. (2008). Identification of genes targeted by CpG island methylator phenotype in neuroblastomas, and their possible integrative involvement in poor prognosis. Oncology.

[62] Coan M., Rampioni Vinciguerra G.L., Cesaratto L., Gardenal E., Bianchet R., Dassi E., Vecchione A., Baldassarre G., Spizzo R., Nicoloso M.S. (2018). Exploring the Role of Fallopian Ciliated Cells in the Pathogenesis of High-Grade Serous Ovarian Cancer. Int. J. Mol. Sci..

[63] Altenberger C., Heller G., Ziegler B., Tomasich E., Marhold M., Topakian T., Müllauer L., Heffeter P., Lang G., End-Pfützenreuter A. (2017). SPAG6 and L1TD1 are transcriptionally regulated by DNA methylation in non-small cell lung cancers. Mol. Cancer.

[64] Chaplin T., Ayton P., Bernard O.A., Saha V., Della Valle V., Hillion J., Gregorini A., Lillington D., Berger R., Young B.D. (1995). A novel class of zinc finger/leucine zipper genes identified from the molecular cloning of the t(10;11) translocation in acute leukemia. Blood.

[65] Yang B., Wang L., Luo X., Chen L., Yang Z., Liu L. (2015). SPAG6 silencing inhibits the growth of the malignant myeloid cell lines SKM-1 and K562 via activating p53 and caspase activation-dependent apoptosis. Int. J. Oncol..

[66] Li X., Yang B., Wang L., Chen L., Luo X., Liu L. (2017). SPAG6 regulates cell apoptosis through the TRAIL signal pathway in myelodysplastic syndromes. Oncol. Rep..

[67] Hengartner M.O. (2000). The biochemistry of apoptosis. Nature.

[68] Jiang M., Chen Y., Deng L., Luo X., Wang L., Liu L. (2019). Upregulation of *SPAG6* in Myelodysplastic Syndrome: Knockdown Inhibits Cell Proliferation via AKT/FOXO Signaling Pathway. DNA Cell Biol..

[69] Lyko F. (2018). The DNA methyltransferase family: a versatile toolkit for epigenetic regulation. Nat. Rev. Genet..

[70] Yin J., Li X., Zhang Z., Luo X., Wang L., Liu L. (2018). SPAG6 silencing induces apoptosis in the myelodysplastic syndrome cell line SKM-1 via the PTEN/PI3K/AKT signaling pathway in vitro and in vivo. Int. J. Oncol..

[71] Yang Y., Wang C., Zhao K., Zhang G., Wang D., Mei Y. (2018). TRMP, a p53-inducible long noncoding RNA, regulates G1/S cell cycle progression by modulating IRES-dependent p27 translation. Cell Death Dis..

[72] Gross J., Angerstein M., Fuchs J., Stute K., Mazurek B. (2011). Expression analysis of prestin and selected transcription factors in newborn rats. Cell. Mol. Neurobiol..

[73] Moon H.M., Wynshaw-Boris A. (2013). Cytoskeleton in action: lissencephaly, a neuronal migration disorder. Wiley Interdiscip. Rev. Dev. Biol..

