# Supplementary material for: The Emerging Role of Sperm-Associated Antigen 6 Gene in the Microtubule Function of Cells and Cancer
Source: Mol Ther Oncolytics. 2019 Sep 10;15:101–7. doi: 10.1016/j.omto.2019.08.011 (PMC6807308; doi:10.1016/j.omto.2019.08.011)
Supplement: Document S1. Figure S1 [file mmc1.pdf]

OMTO, Volume *15*

Supplemental Information

The Emerging Role of Sperm-Associated Antigen 6 Gene in the Microtubule Function of Cells and Cancer

Da-Fang Zheng, Qi Wang, Jing-Ping Wang, Zheng-Qi Bao, Shi-Wu Wu, Li Ma, Da-Min Chai, Z. Peter Wang, and Yi-Sheng Tao

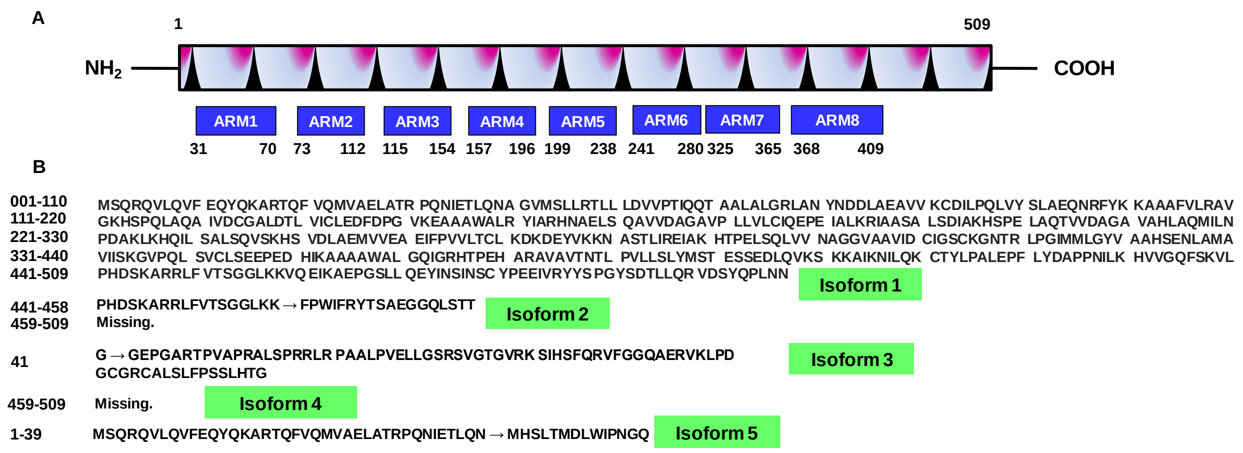

Supplementary figure 1: The structure and the sequences of SPAG6 are illustrated. A, SPAG6 protein has eight ARM domains. B, SPAG6 protein has five isoforms with different sequences.
